# Research strategies of the N-peptide fusion inhibitor: a promising direction for discovering novel antivirals

**DOI:** 10.1128/jvi.02289-24

**Published:** 2025-04-10

**Authors:** Yan Huang, Guodong Liang, Taoran Wang, Yuheng Ma, Lu Ga, Lijun Sun, Xiao Qi, Wei Zhang, Ruijuan Li, Yan Zhao, Zhao Meng, Xin Gao

**Affiliations:** 1 Key Laboratory for Candidate Medicine Design and Screening Based on Chemical Biology, College of Pharmacy, Inner Mongolia Medical University 66287 , Hohhot, China; 2 Beijing Institute of Pharmacology and Toxicology 96705 , Beijing, China; New York University Department of Microbiology, New York, New York, USA

**Keywords:** virus, research strategies, antivirals, N-peptide fusion inhibitor

## Abstract

AIDS, caused by HIV-1, is a devastating condition that severely compromises the human immune system, often resulting in fatal consequences. The primary therapeutic approach for AIDS involves a combination of multiple agents, known as “cocktail therapy,” aimed at maximizing and sustainably suppressing viral replication within patients. The ongoing discovery of novel compounds and the establishment of innovative research strategies have become the mandatory path to provide increasingly effective treatment options for AIDS. Peptide-based fusion inhibitors, exemplified as enfuvirtide, are able to target the six-helix bundle fusion core in HIV-1 envelope protein and function during the early stage of viral invasion. However, the prolonged and intensive use of enfuvirtide in clinical settings has posed significant challenges, including the emergence of drug resistance. N-peptide fusion inhibitors, whose sequences are different from enfuvirtide, exhibit potential anti-HIV-1 activity and inhibition of drug-resistant strains through the advanced coiled-coil conformation and are expected to serve as novel peptide inhibitors in the iteration of enfuvirtide. This paper provides a comprehensive summary of N-peptide fusion inhibitor research and development (R&D) to date, with the aim of providing investigators with prospective ideas for exploring antivirals.

## INTRODUCTION

As is known to all, the human immunodeficiency virus (HIV) could attack CD4^+^ T lymphocytes in the immune system, which can lead to infection with a variety of diseases (1). HIV is classified into HIV-1 and HIV-2 based on genetic differences, and the predominant strain currently prevalent worldwide is the HIV-1 strains. In 1981, scientists identified the first case of acquired immunodeficiency syndrome (AIDS) caused by HIV-1, subsequently proven to be a highly lethal viral infection (2). Until today, the latest “2023 Progress Report on the Global AIDS Response - The Path to an End” published by the United Nations Programme on HIV/AIDS (UNAIDS) (3) shows that AIDS has become a chronic infectious disease that can be controlled, but not fully cured. Meanwhile, according to the UNAIDS survey, by 2023, there will still be more than 39 million people living with HIV. Although UNAIDS has set the ambitious goal of ending AIDS as a public health threat by 2030 in alignment with the Sustainable Development Goals (SDGs), the global HIV/AIDS epidemic remains a critical challenge. The development of novel drugs, especially those targeting drug-resistant strains and latent viral reservoirs, remains an indispensable priority in the fight against HIV/AIDS.

**Fig 1:**
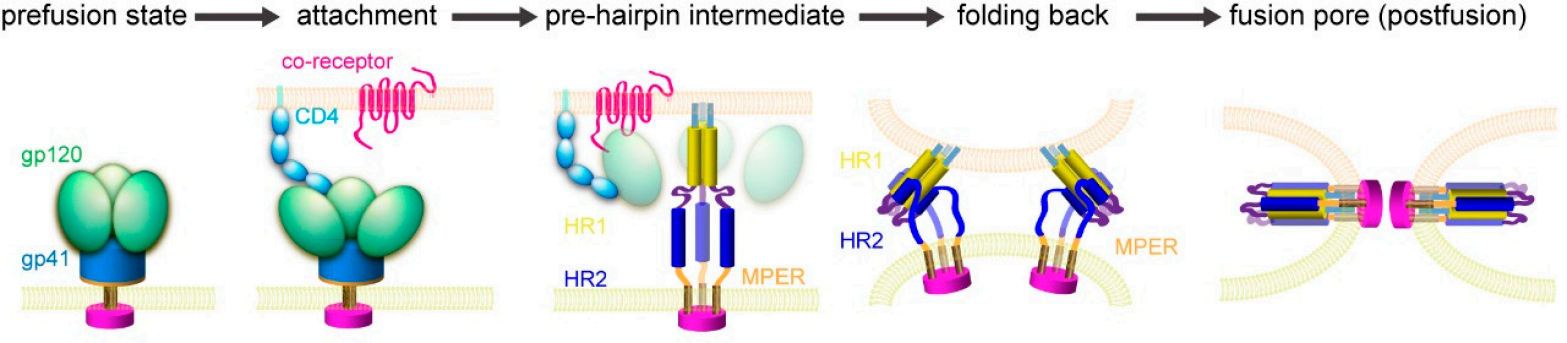
Membrane fusion process of HIV invading host cells (8).

The primary treatment for AIDS is highly active antiretroviral therapy (HAART), also referred to as “cocktail therapy,” which involves the simultaneous administration of three to four antiretroviral drugs, aiming to maximize the suppression of HIV-1 replication and reduce its viral load. Due to the different action mechanisms of multiple medicines, it is difficult for the virus to develop resistance to all medicines at the same time, thus maintaining therapeutic effectiveness for a longer time (4). Antiviral medicines currently in use include nucleoside/nucleotide reverse transcriptase inhibitors (NRTIs), non-nucleoside reverse transcriptase inhibitors (NNRTIs), protease inhibitors (PIs), integrase inhibitors (INSTIs), chemokine receptor antagonists (CRAs), adhesion inhibitors (AIs), and fusion inhibitors (FIs). Of these, fusion inhibitors are effective at the stage of viral invasion into host cells, and listed agents show advantages in fast onset of action, high safety, strong efficacy, and low metabolic drug–drug interactions (5). Therefore, fusion inhibitors show great potential toward further exploitation. HIV-1 invasion into host cells is mainly based on the membrane fusion process (Fig. 1). First, HIV could recognize and bind to the host cell surface receptor, then trigger conformational change of envelope proteins. The N-terminal fusion peptide of transmembrane subunit in envelope proteins is then anchored to the host cell membrane. Afterward, the key N-terminal heptads repeat (NHR, or HR1) region of the transmembrane subunit is spontaneously assembled to form the coiled-coil structure (N-trimer), and the downstream C-terminal heptads repeat (CHR, or HR2) region immediately folds backward to interact with the NHR region, eventually forming the stable six-helix bundle (6-HB) (6). Then, HIV genetic material enters into the host cell through the fusion core. Theoretically, blocking the HIV-1 membrane fusion process is an effective strategy against viral infection; the 6-HB structure is an ideal target. Peptide-based fusion inhibitors could target the 6-HB structural domain and prevent virus-host cell membrane fusion (7).

Customarily, peptide-based fusion inhibitors are categorized as C-peptide and N-peptide on the basis of sequence origins and different binding targets that prevent 6-HB formation. Currently, enfuvirtide (T20) is the first marketed C-peptide fusion inhibitor against HIV (9, 10). However, in the clinical application process, T20 has several shortcomings such as a high level of resistance development, short half-life *in vivo*, and rapid renal clearance, which significantly limits the clinical application (11–13). Therefore, it is necessary for researchers to search for novel fusion inhibitors that can address the T20 clinical application problems. However, researchers are currently focusing on mainly C-peptides derived from the HIV-1 envelope CHR region, which still cannot compensate fully for the T20 shortcomings (14–17). The N-peptide fusion inhibitor, with a sequence entirely distinct from the listed peptide segments, originates from the N-terminal repeat sequence of the HIV-1 envelope protein gp41 and interacts with the CHR region downstream of the NHR region to disrupt the endogenous 6-HB structure through coiling, thereby preventing virus-target cell membrane fusion and exhibiting notable antiviral activity (18, 19). Researchers have developed N-peptide fusion inhibitors with higher biological activity and metabolic stability by employing strategies such as amino acid substitution and heterologous peptide bond crosslinking, demonstrating good inhibitory activity in experiments. Importantly, the improved stability and high inhibition to drug-resistant strains of N-peptides offer the future solutions to the current clinical dilemma as drug candidates (20). With the deepening research into the fusion mechanism of HIV-1 and the development of novel N-peptide fusion inhibitors, there is potential for achieving more efficient and broader-spectrum anti-HIV therapies in the future, providing a theoretical basis for the optimized design of targeted peptide inhibitors. This article summarizes the N-peptide research progress and development strategies to date, aiming to provide crucial guidance for the discovery of novel anti-HIV-1 agents.

## BIOACTIVE CONFORMATION FOR N-PEPTIDE FUSION INHIBITORS: 1α-HELIX OR 3α-HELICES?

**Fig 2:**
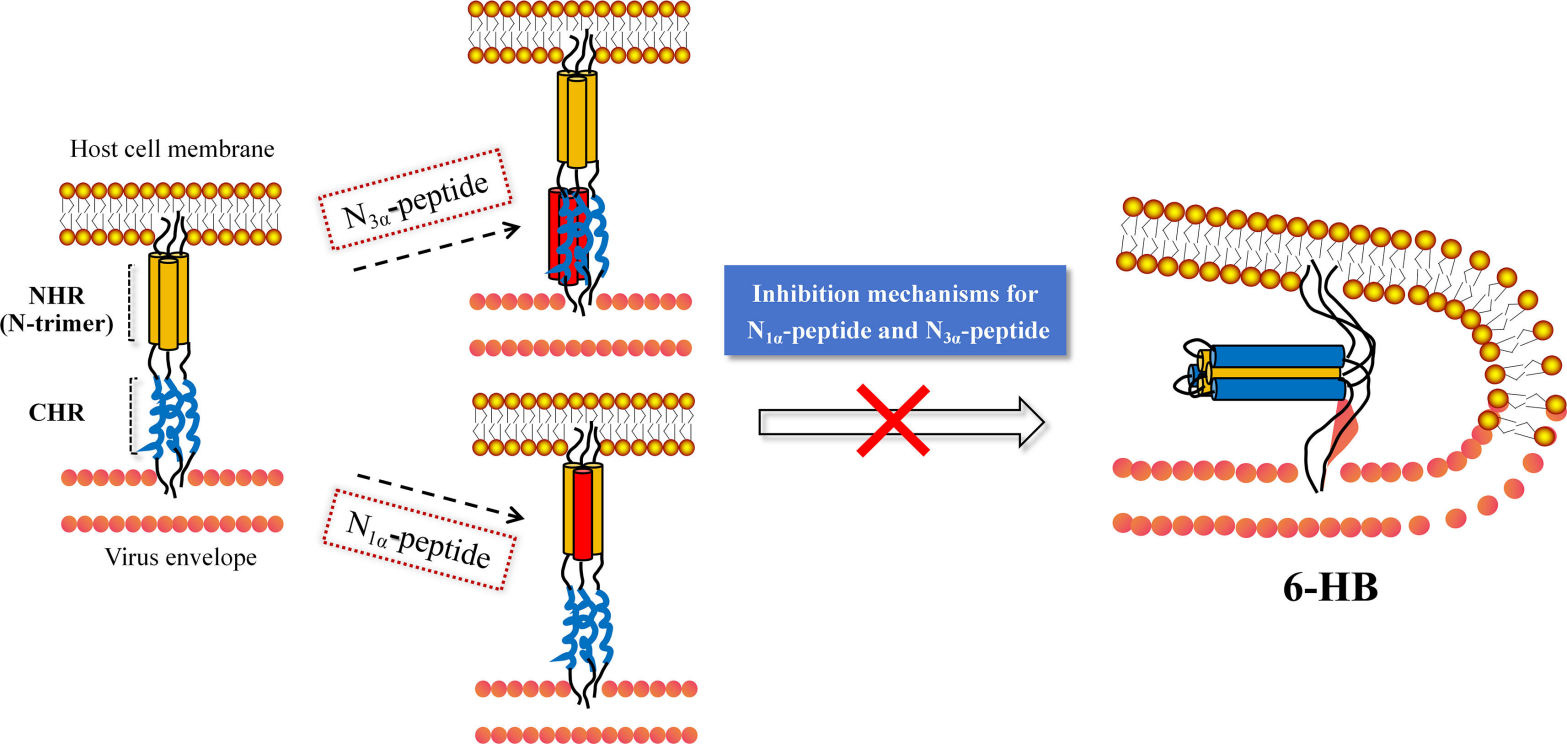
N_1α_ interacts at NHR.

Previous studies demonstrated that the N-peptide could inhibit HIV-1 membrane fusion via mainly two mechanisms (Fig. 2) (19, 21). One mechanism is that N-peptide as 1α-helix (we called N_1α_-peptide) could admix into natural NHR region assembly, forming heterologous N-trimer to prevent NHR region→6-HB deformation process; another mechanism is that N-peptide as 3α-helices self-assembly (called N_3α_-peptide) could interact with the natural CHR region to disrupt CHR region→6-HB deformation process. The above mechanisms bring up a key issue: whether to design potential N-peptide fusion inhibitors with a 1α-helix or a 3α-helices as the active structure? Notably, under physiological or, e.g., phosphate-buffered saline (PBS) solution conditions, isolated natural N_1α_-peptides are less stable, prone to aggregation and precipitation, and unable to bind targets as the 1α-helix conformation, and thus have lower antiviral activity than C-peptides. In contrast, the N_3α_-peptides were able to mimic the coiled-coil conformation (N-trimer) during the deformation of the NHR region to 6-HB and showed significant antiviral activity with high fitness to the target (22). Currently, researchers have successively discovered many pioneer N_3α_-peptides and established a wealth of modification research strategies, mainly including a site-mutagenesis strategy for assembling the coiled-coil bioactive conformation, a self-assembly strategy with chimeric tool peptides, a strategy for small-molecule skeleton stapled N-multimers, a strategy for constructing covalent bond among coiled-coil helices, and so on. The N_3α_-peptides obtained through the above strategies essentially showed high stability, nanomolar-level antiviral activity, potential ability against drug-resistant strains, and good pharmacokinetic characteristics, which are suitable as antiviral candidates for further discovery.

## SITE-MUTAGENESIS STRATEGY FOR ASSEMBLING THE COILED-COIL BIOACTIVE CONFORMATION

**Fig 3:**
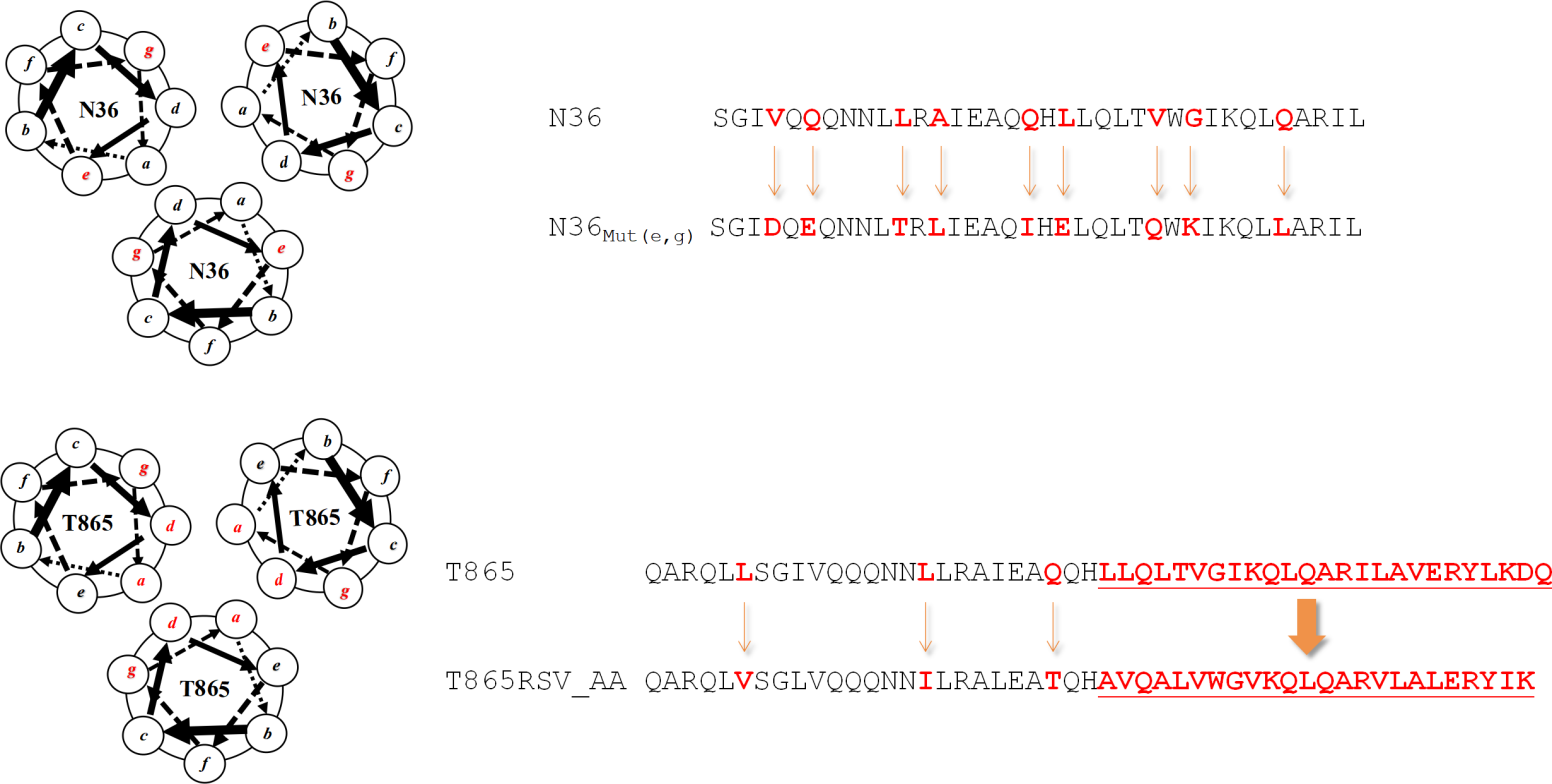
Sequence of designed N-peptides based on site-mutagenesis strategy.

As the commonly utilized approach for structure optimization, the site-mutagenesis strategy holds a pivotal position in the peptide-based R&D realm. In the peptide body, all amino acid residues with differentiated side chain motifs and physicochemical properties together determine the overall conformation and biological activity. The site-mutagenesis strategy precisely utilizes the aforementioned principle, achieving fine-tuned regulation of peptide structure and function through precise modulation of specific amino acid residues (23). The site-mutagenesis strategy precisely regulates the structure and function of peptides by replacing specific amino acid sites. Applied to the N_3α_-peptides investigation, regular site mutations enable the assembly of N-trimer mimics conformation. In 2002, Bewley et al. (24) employed the natural N36 as a template and introduced mutations at the “e” and “g” positions of its amino acid residues, resulting in N36_Mut(e, g)_ (Fig. 3). Analytical ultracentrifugation and circular dichroism (CD) spectroscopy confirmed that N36_Mut(e, g)_ can self-assemble into an N-trimer structure and effectively inhibit HIV-1 infection (half maximal inhibitory concentration [IC_50_] = 308 nM). In order to further investigate the site mutation design pattern, Dwyer et al. (25) replaced the isoleucine residue at the “*g*” position in T865 with alanine residue, and then boldly replaced the residue of “*a, d*” sites with the residue originated from the natural convoluted helix of respiratory syncytial virus (Fig. 3), and then the substituted T865RSV_AA unexpectedly showed a very high stability and enhanced potency against HIV-1. The results discussed above not only verify the effectiveness of the site-mutagenesis strategy in forming N-peptide coiled-coil and improving the inhibitory activity, but also lay the foundation for self-assembly strategy with chimeric tool peptides, constructing covalent bonds among coiled-coil helices, or other strategies. Moreover, the site-mutagenesis strategy has shown great potential for peptide design against other viruses, such as influenza viruses (IAVs) and severe acute respiratory syndrome coronavirus 2 (SARS-CoV-2) (26–28).

## SELF-ASSEMBLY STRATEGY WITH CHIMERIC TOOL PEPTIDES

**Fig 4:**
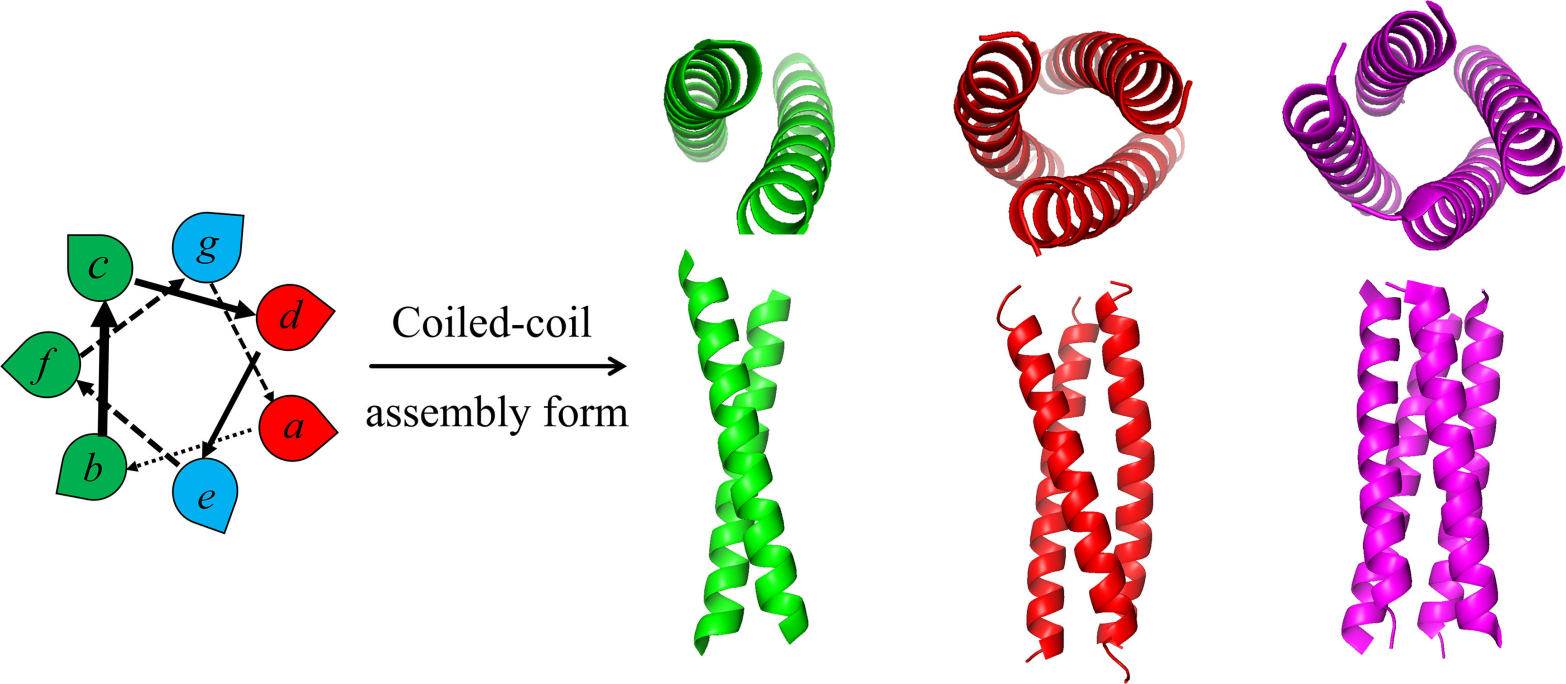
Common assembly forms of the coiled-coil.

The coiled-coil structure is a superhelical structure found in many natural proteins and has become an ideal model for protein research as repetitive and regular structure (29). In 1953, Crick et al. (30) first proposed the coiled-coil structure, as a superhelical structural domain, which consists of two or more α-helices twisted parallel or antiparallel to each other through hydrophobic interactions. The peptide sequences that compose the coiled-coil structure are presented as having the heptad-repeat principle, with each repeat sequence region generally containing seven amino acid residues, known as the heptad-repeat (HR) region (Fig. 4). In the HR region, amino acid residue sites are labeled with “*a, b, c, d, e, f, g*” in order, in which the “*a, d*” sites are mostly hydrophobic amino acid residues, such as leucine residues, isoleucine (Ile) residues, etc., which hydrophobically assemble the coiled-coil core; the “*e, g*” sites are located on the outside of the coiled-coil core, and most of them are polar charged amino acid residues, such as lysine (Lys) residue, glutamic acid (Glu) residue, etc., which can maintain the stability of coiled-coil structure through electrostatic interaction at the outer side (31).

**Fig 5:**
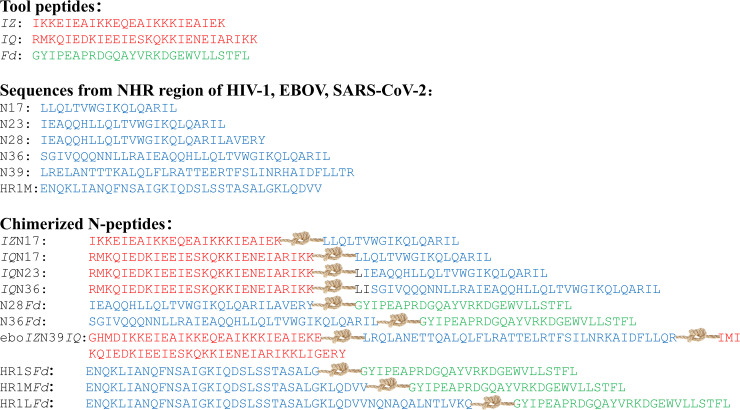
Assigned NHR sequences of different viruses (cords represent tool peptides and key sequences chimerized by covalent bond).

The coiled-coil assembly method can improve protein/peptide stability, whereas natural N-peptides after isolation cannot self-assemble to form a 3α-helical structure. In view of the above complementary and synergistic properties, the researchers attempted to chimeraize coiled-coil tool peptides’ natural N-peptide sequences in the hope of obtaining novel N-peptides with potential antiviral activity. In 1998, Suzuki et al. (18) designed a tool peptide with four heptad repeats, named *isoleucine zipper* (*IZ*). In the *IZ* sequence, Ile residues are located in “*a*” and “*d*” positions for assembling coiled-coil in the hydrophobic manner. Subsequently, Glu and Lys residues are located in “*e*” and “*g*” positions for creating stable electrostatic interactions among helices, and Glu and alanine residues are fixed at “*b*” and “*c*” positions. Ultimately, the *IZ* sequence is finalized as *YGG(IEKKIEA)_4_*. Experimentally, *IZ* was demonstrated to be an ideal tool peptide capable of assisting in the coiled-coil formation with the N17 sequence from the HIV-1 NHR region, and the combined *IZ*N17 exhibited nanomolar level anti-HIV-1 activity and superior thermal stability. In 2001, Eckert et al. (19, 32) obtained another tool peptide with the sequence *RMKQIEDKIEEILSKQYHIENEIARIKKLIGER*, named *IQ*, which could assemble into 3ɑ-helices the same as *IZ. IQ* could embed into N17, N23, and N36 sequences from the HIV-1 NHR region, yielding a series of novel N-peptides, called *IQ*N17, *IQ*N23, and *IQ*N36 (Fig. 5), which also exhibit nanomolar level anti-HIV-1 activity. The aforementioned examples demonstrate that tool peptides provide remarkable supplementary functionalities for N-peptides to effectively exert antiviral activity. Besides *IZ* and *IQ*, Yang and Chen et al. (20, 33, 34) discovered the third tool peptide, named *Fd*, with sequences from Foldon protein in T4 phage fibronectin (Fig. 5). The anti-HIV-1 experiment results indicated that chimeric N-peptides, named N28Fd and N36Fd, were 99 nM and 39 nM, respectively, and antiviral potency aligned with the previously mentioned observed trend: the anti-HIV-1 activity of natural N-peptide stays at the micromolar level, whereas the anti-HIV-1 activity of the chimeric N-peptide could reach the nanomolar level.

Chimeric tool peptides such as *IZ, IQ,* and *Fd* exhibit broad potential applications in viral fusion inhibitors, not only in the HIV-1 field but also in other virus studies. Drawing inspiration from the anti-HIV-1 N-peptide designing, Clinton et al. (35) innovatively constructed the anti-Ebola virus (EBOV) N-peptides by embedding the coiled-coil tool peptides *IQ* and *IZ* in the EBOV N-peptide C-terminus, named ebo*IZ*N39*IQ* (Fig. 5), with stable coiled-coil structure, based on the similarities in fusion mechanisms between EBOV and HIV-1. The ebo*IZ*N39*IQ* not only successfully mimicked the target helical structure but also exhibited exceptional thermal stability. To further confirm whether ebo*IZ*N39*IQ* can adopt the active conformation N-trimer in the pre-hairpin intermediate, the researchers conducted detailed studies on the binding of ebo*IZ*N39*IQ* to native target using surface plasmon resonance analysis techniques. The results demonstrated tight binding affinity with 14 nM dissociation constant between the native target and ebo*IZ*N39*IQ*, comparable to the interaction strength of HIV-1 N-peptides and native target, thereby strongly evidencing effective presentation of the ebo*IZ*N39*IQ* N-trimer structure. Similarly, aiming to develop peptide fusion inhibitors against SARS-CoV-2, Bi et al. (36) fused the trimerization motif Fd to SARS-CoV-2 NHR-derived peptide, finally obtaining novel N-peptides (Fig. 5). These N-peptides (named HR1S*Fd*, HR1M*Fd,* and HR1L*Fd*) indeed formed stable trimers and showed dramatically increased antiviral activity and thermostability compared with the natural HR1 from the NHR region. Moreover, HR1M*Fd* showed broad-spectrum inhibitory activity against various SARS-CoV-2 pseudoviral mutants, SARS-CoV pseudovirus, and Middle East respiratory syndrome coronavirus (MERS-CoV) pseudovirus.

Therefore, based on the self-assembly strategy with chimeric tool peptides, the novel N-peptide could self-assemble into 3α-helices structure and thus bind tightly to the target, significantly improving stability, antiviral activity, and viral mutation adaptability. However, the current small variety of tool peptides is far from matching the lead compound, and more tool peptides are needed to support the development of candidate antivirals in the future.

## STRATEGY FOR SMALL-MOLECULE SKELETON STAPLED N-MULTIMERS

**Fig 6:**
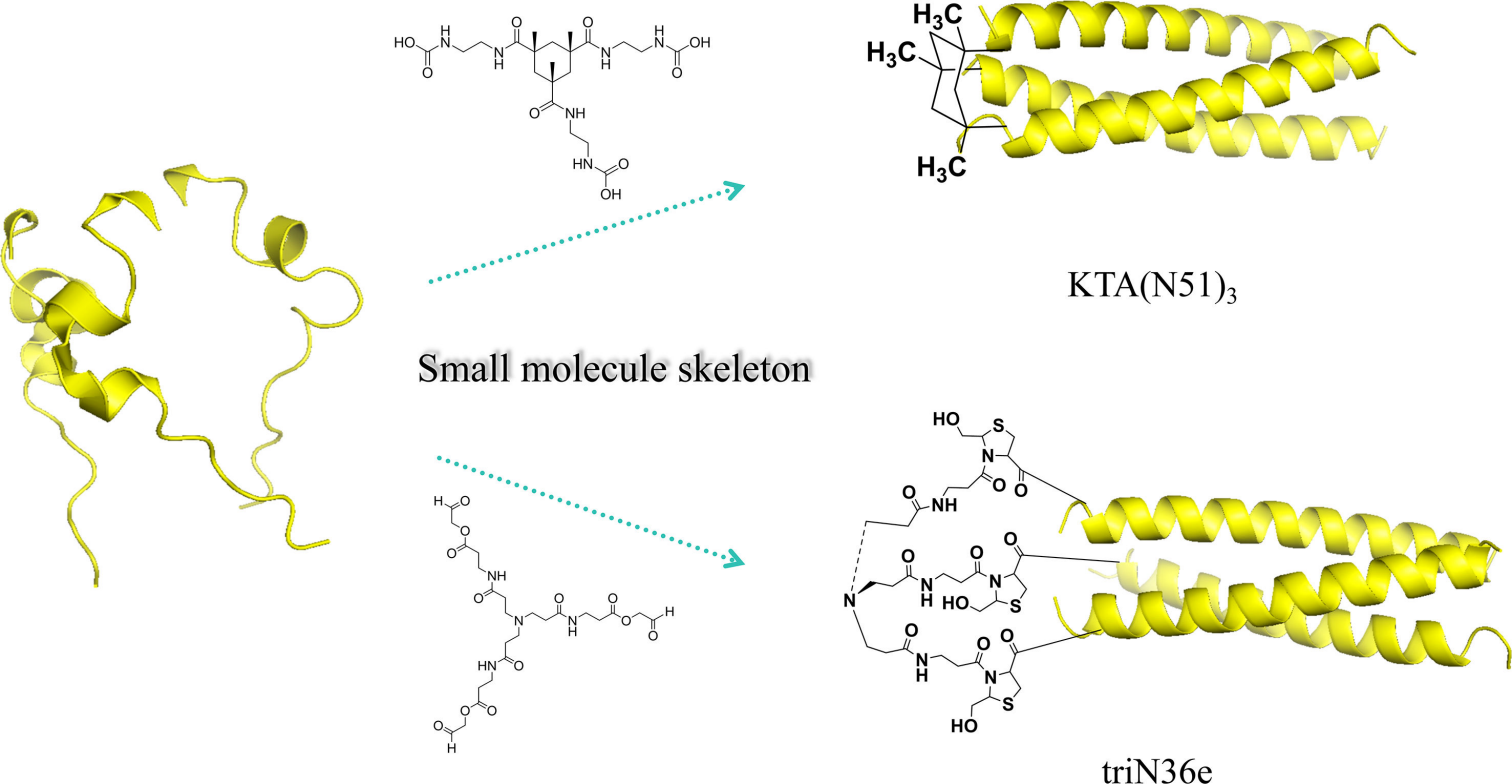
Schematic diagram of small-molecule skeleton stapled N-multimers.

Currently, the small-molecule skeleton plays a crucial role in biochemistry and drug design, as they could not only serve as the cornerstone for stable structures, but also endow new functions and characteristics through specific chemical modifications (37). In the fusion inhibitors field, small-molecule skeletons are the crucial design tool with plasticity and modifiability, and researchers developed a method to staple N-multimers forming 3α-helices structure through small-molecule skeleton rigidity to exert antiviral efficiency. In 2010, Nakahara et al. (38) connected a novel small-molecule skeleton with three equal-length branching junctions with ester aldehyde groups to the N-terminal cysteine of the natural peptide N36 in aqueous solution and finally obtained the novel N-peptide, named triN36e (Fig. 6). CD experiments revealed that triN36e possesses ɑ-helix structure, with significantly higher helix content compared to N36. In the anti-HIV-1 experiment, compared with N36, triN36e showed more than threefold increase in antiviral activity and a 30-fold increase in binding capacity, demonstrating the feasibility of small-molecule skeleton stapled N-multimers strategy. In 2024, Wu et al. (39) used Kemp’s triacid (KTA) skeleton to immobilize natural peptide N51 to get trimeric peptide KTA(N51)_3_ (Fig. 6), which exhibited 10-fold enhancement in the inhibitory activity compared to N51. In order to explore the generalizability of the small-molecule skeleton strategy, this team prepared tribromoacetylated scaffold, linking to N51, yielding a series of novel N-peptides, all of them possessing effective antiviral activities and virus mutant adaptability. It is worth mentioning that the small-molecule skeleton stapled multimers strategy can also be applied in the C-peptide fusion inhibitors. Nomura’s team (40) obtained a novel C-peptide triC34e by selectively coupling C3 template to natural peptide C34, which has 100-fold more antiviral activity than single C34. Based on the small-molecule skeleton stapled N-multimers strategy, it is possible to rigidly constrain three homologous natural N-peptides to get one macromolecule; the small-molecule skeleton is positioned at the N-peptide terminus, allowing N-peptide to assemble into coiled-coil structure with high freedom degrees. In addition, this strategy may play a broad role in the fusion inhibitor investigation such as C-peptide.

## STRATEGY FOR CONSTRUCTING COVALENT BOND AMONG COILED-COIL HELICES

**Fig 7:**
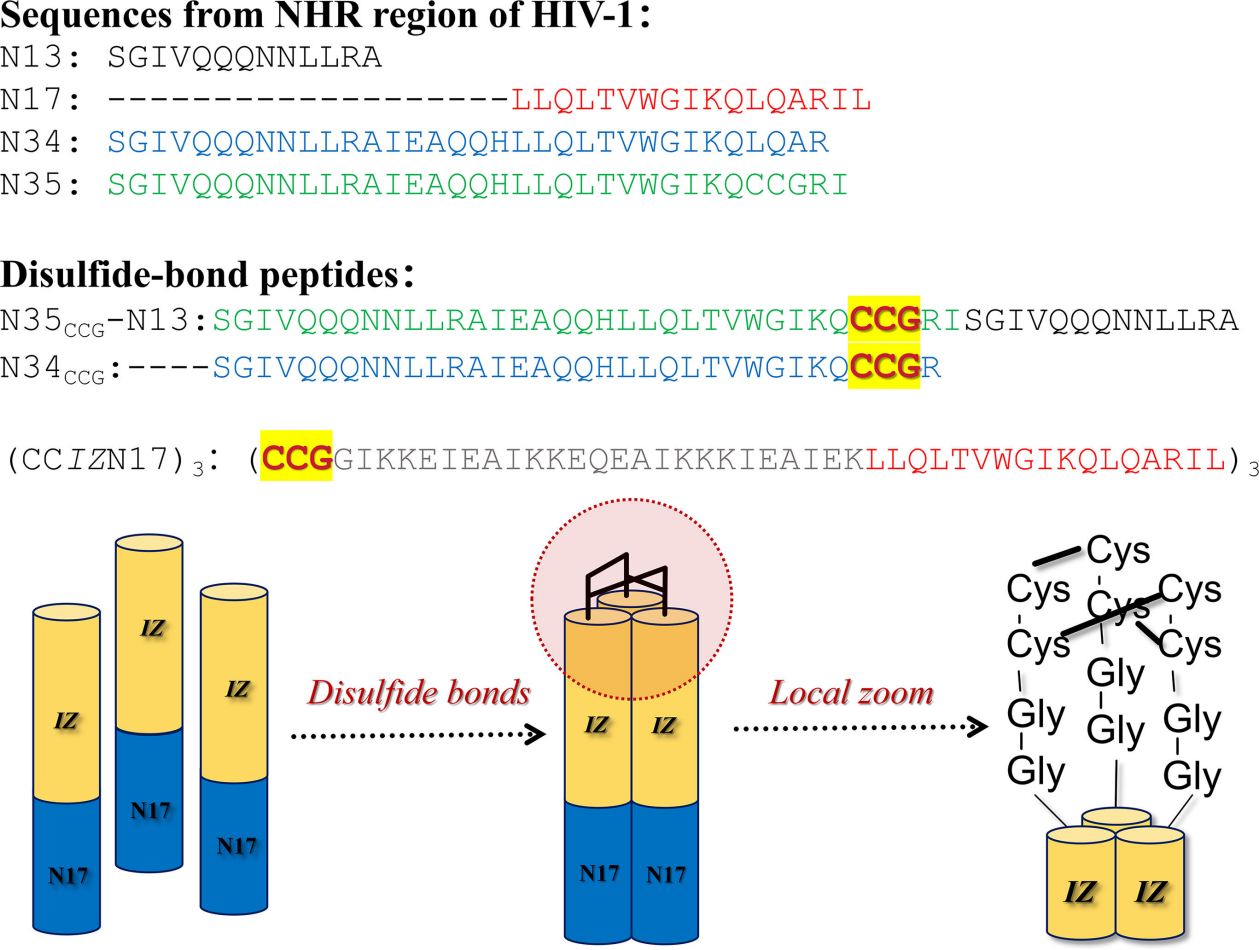
Schematic diagram of disulfide bond stapled N-peptides.

By comparison with the C-peptides derived from the CHR region, peptides derived from the NHR region generally require the construction of N-trimer mimetics to exhibit potential inhibitory effects. Chemistry-driven covalent bond bundling coiled-coil provides another well-established approach for N-peptide designing. The introduction of covalent bonds not only prefolds the N-peptide to minimize the conformational entropy loss when interacting with the CHR region, but also improves the thermal stability, protease stability, and *in vitro* metabolic stability of the N-peptide (41). Disulfide bonds, as covalent linkages, are extensively utilized in protein/peptide design. Disulfide bonds can form rigid lock-arm structures within or between protein/peptide molecules, thereby significantly enhancing structural integrity and thermal stability. Given these advantages, researchers have introduced disulfide bonds into N-peptides to stabilize their active conformations (42). Louis et al. (43) firstly introduced cysteine residues at specific positions in the N-peptide sequences to establish interchain disulfide bonds by oxidation reaction and ultimately obtained N35_CCG_-N13 and N34_CCG_ with disulfide bond arm (Fig. 7), which inhibited HIV-1 envelope protein-mediated cell-cell fusion at nanomolar level. Considering that the antiviral activity of *IZ*N17 is somewhat limited by the self-association equilibrium, Bianchi et al. (44) introduced cysteine residues at the *IZ*N17 sequence terminus, which self-associated into the coiled-coil in solution, and the interhelical cysteine residues were also oxidized to form disulfide bonds, finally obtaining a novel N-peptide named (CC*IZ*N17)_3_ (Fig. 7). Experimental results showed that (CC*IZ*N17)_3_ exhibited remarkable thermodynamic stability with *T*_m_ >90°C in 2 M GdnHCl solution (compared to 61.5°C for *IZ*N17), and inhibitory activity against HIV-1 reached 40 pM–380 pM. In addition, (CC*IZ*N17)_3_ was further efficacious in neutralizing acute viral infections in peripheral blood mononuclear cells and exhibited a broad spectrum of HIV-1, including R5, X4, and R5/X4 strains. Overall, the anti-HIV-1 potency of (CC*IZ*N17)_3_ exceeded that of T20 as well as monomeric *IZ*N17. The above findings mean that disulfide bond for bundling coiled-coil structure is a feasible strategy, not only because of the relatively easy synthetic method but also because of the substantial improvement in stability and activity of novel N -peptides. It is worth noting that disulfide bonds also have certain drawbacks, such as being easily degraded by glutathione reductase or oxidoreductase, which may affect the N-peptide activity *in vivo* to some extent. Nevertheless, disulfide bonds still provide promising ideas for N-peptide design (45).

The isopeptide bond, as a specialized covalent bond present in bacterial hyphae, consists of the non-alpha amino or carboxyl group of an amino acid condensed with the carboxyl or amino group of another amino acid. The isopeptide bond is able to significantly maintain the bacterial hyphae integrity when the bacteria are exposed to severe mechanical, thermal, and protein hydrolysis stress conditions. In comparison to disulfide bonds, isopeptide bonds have higher thermodynamic stability, chemical stability, and pH tolerance. These excellent properties make isopeptide bonds a handy tool for protein structure modification, especially for the N-peptides with poor physical and chemical stability and requiring coiled-coil active conformations (46–49).

**Fig 8:**
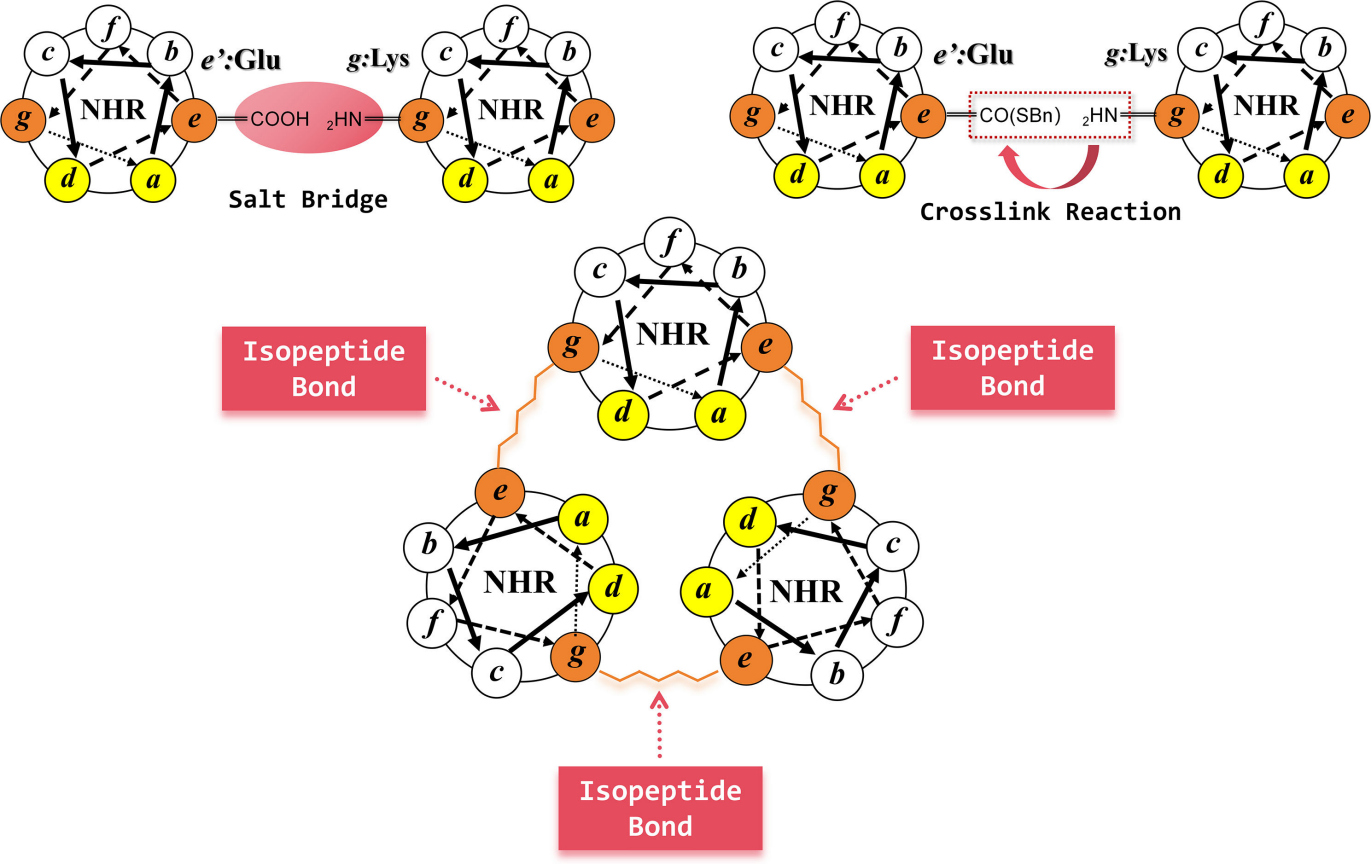
Schematic diagram of isopeptide bond stapled N-peptides.

Wang et al. (22) pioneered the innovative application of isopeptide bonds to N-peptide. First, Wang et al. designed the tool peptides 3HR and 4HR (consisting of the sequences of *IQQIEQK IHHIEQR IQQIEQR IQQIEQR* aligned from “*a*” to “*g*” positions), which are capable of self-assembling into triple helices, and combined with natural peptide sequences derived from the HIV-1 NHR region to form the N-peptide primary sequences. Spatially, the carboxyl group of the Glu residue at the “*e”* position in one helical peptide could form an intermolecular salt bridge with the amino group of the Lys residue at the “*g”* position in the neighboring helical peptide, which provides a precondition for the isopeptide bond creation. Then, Wang et al. skillfully designed the Glu carboxyl thioesterification at the one-helix “*e*” position, forming an isopeptide bond with the Lys amino group at another-helix “*g*” position in PBS solution by acyl transfer reaction (Fig. 8). The isopeptide-bonded N-peptides were not completely denatured at 90°C in stability experiments and were comparable to T20 in anti-HIV-1 activity. As reported in another publication (50), this same group successfully synthesized another isopeptide-bonded N-peptide based on natural NHR sequences by using N36 as the lead compound and introducing isopeptide bonds with site mutation and thioesterification modifications. These novel N-peptides also exhibited excellent physical and chemical stability and anti-HIV-1 activity. Subsequently, many researchers have continued to delve deeper and deeper to elucidate the isopeptide bond formation rules.

**Fig 9:**
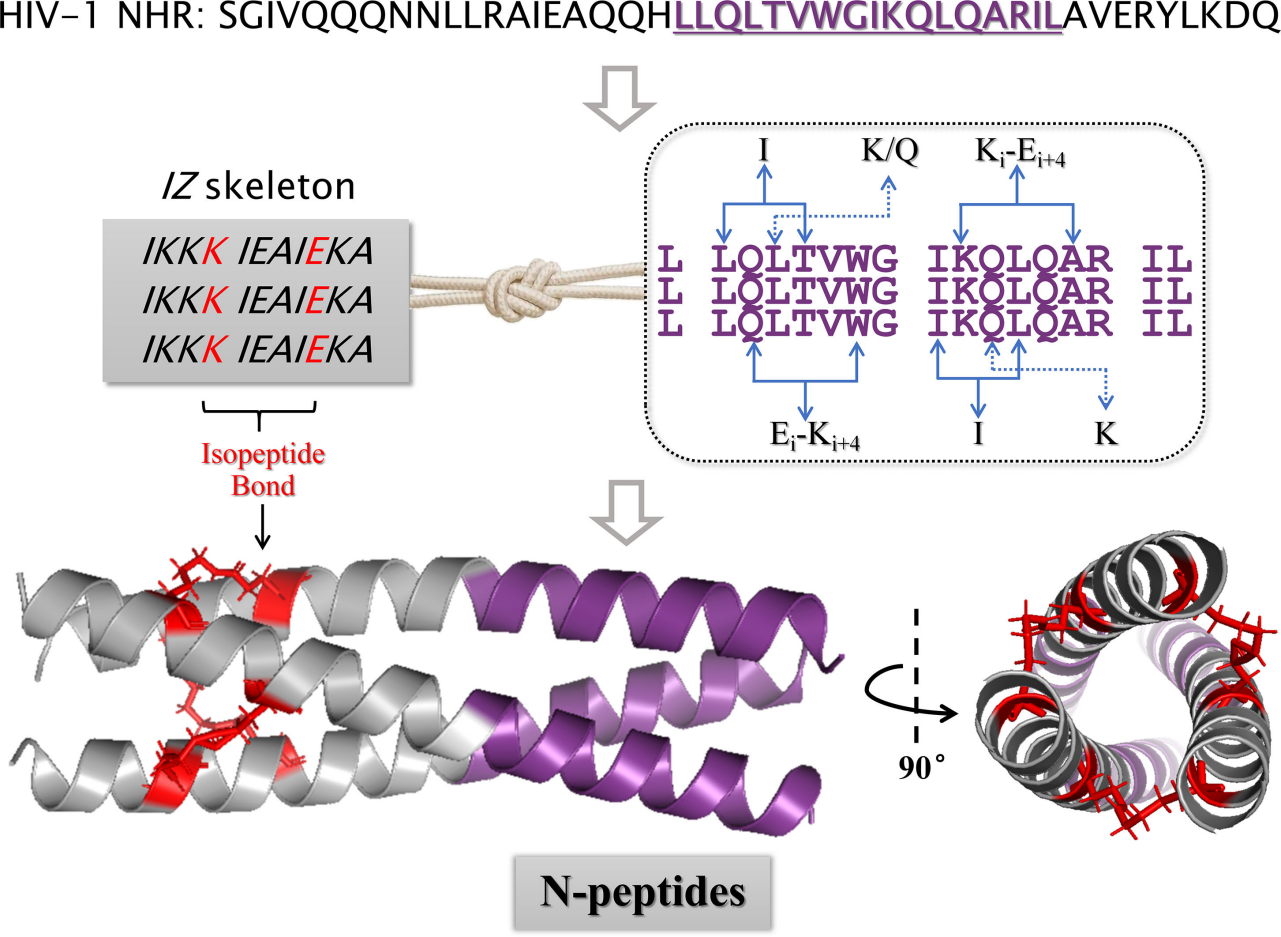
Schematic N-peptide design based on constructing isopeptide bonds (52).

Wang et al. (51) further investigated the applied isopeptide bonds to N-peptides and systematically summarized three major rules of isopeptide bond bundling coiled-coil. (i) Isopeptide bond construction at tool peptides at different sites: Wang et al. used IZN17 sequence as a template and obtained (*IZ*N17L)_3_ by cross-linking the Glu at the *4* position of one helix with the Lys at the *9'* position of another adjacent helix to form an isopeptide bond; obtained (*IZ*N17M)_3_ by cross-linking the Glu at the *4+7* position of one helix with the Lys at the *9'+7'* position of another adjacent helix; obtained (*IZ*N17R)_3_ by cross-linking the Lys at the *4+7+7* position of one helix with the Glu at the *9'+7'+7'* position of another adjacent helix. In summary, the Glu residues at *4*, *4+7*, and *4+7+7* positions within the tool peptide sequence could react with the Lys residues at *9'*, *9'+7'*, and *9'+7'+7'* positions in the parallel sequence, respectively, leading to the formation of three pairs of isopeptide bonds among helices. (ii) Truncating tool peptides: since the tool peptide only acts as an assembly role, it can be shortened in terms of the synthesis difficulty and production cost considerations while ensuring the N-peptide antiviral ability. Wang et al. selected (IZN17R)_3_ as a template and shortened 7, 10, and 14 amino acid residues from the IZ N-terminal, respectively, and were still able to construct isopeptide bonds and obtain (IZ17N17)_3_, (IZ14N17)_3_, and (IZ10N17)_3_ with nanomolar anti-HIV-1 activity. (iii) Extending peptide sequences from the natural NHR region: to further enhance the binding affinity toward the target and inhibitory activity of N-peptides, it is necessary to increase the natural sequence length in N-peptide primary structure. Wang et al. selected the shorter peptide (*IZ*14N17)_3_ and (*IZ*10N17)_3_ as templates and extended seven natural amino acid residues at the N17 N-terminal and C-terminal to obtain (IZ14N24N)_3_, (IZ10N24N)_3_, (IZ14N24C)_3_, and (IZ10N24C)_3_. All of these showed higher antiviral activity and stability compared to the positive control. On the basis of the above studies, our team even achieved artificial N-peptides by employing isopeptide bonds (Fig. 9) (52). The most active compound, IZNP02QE, surpassed the positive control by demonstrating remarkable nanomolar-level inhibitory activity against HIV-1.

It is promising that the isopeptide bond bundling coiled-coil strategy can also be applied to the development of other viral fusion inhibitors. Zheng et al. (21) selected a 21-amino-acid residue fragment from the NHR region of MERS-CoV S2 subunit as a template and constructed an N-trimer structural model by forming isopeptide bonds. Compared with unmodified monomer, the novel N-peptide did not show the aggregation and had stronger thermal stability, and the anti-MERS-CoV activity was comparable to that of positive control. However, the current synthesis process of isopeptide bond is relatively complicated, which limits N-peptide development to some extent. Therefore, the synthetic process needs to be continuously explored and optimized in the future to provide more powerful support for N-peptides.

## OTHER STRATEGIES

The biotechnological recombinant technique has achieved “protein/peptide custom manufacturing” by enabling DNA precise manipulation (53). The researchers were able to translate endogenous NHR sequences into proteins by DNA translation, which were able to spontaneously assemble as a multimeric form (containing N-trimer), thus exhibiting significant antiviral capabilities. Louis et al. (43, 54) first successfully obtained the N35CCG-N13 protein by using recombinant technology, which consists of natural N35 and N13 forming N-trimer mimics structure by head-to-tail linkage. Louis et al. further skillfully ligated N36 and N35, originating from the HIV-1 NHR region, with C28 originating from the CHR region and yielded the recombinant protein NCCG-gp41, which exhibited the potent inhibitory ability by HIV-1 envelope-mediated cell-cell fusion (IC_50_ = 16.1 nM), flush with the control C34. Utilizing biotechnological recombinant techniques, the N-trimer structure construction has also been achieved. Based on the ability of NHR and CHR to form six-advanced helix bundles, the design of supercoiled macromolecules beyond the N-trimer is also possible.

**Fig 10:**
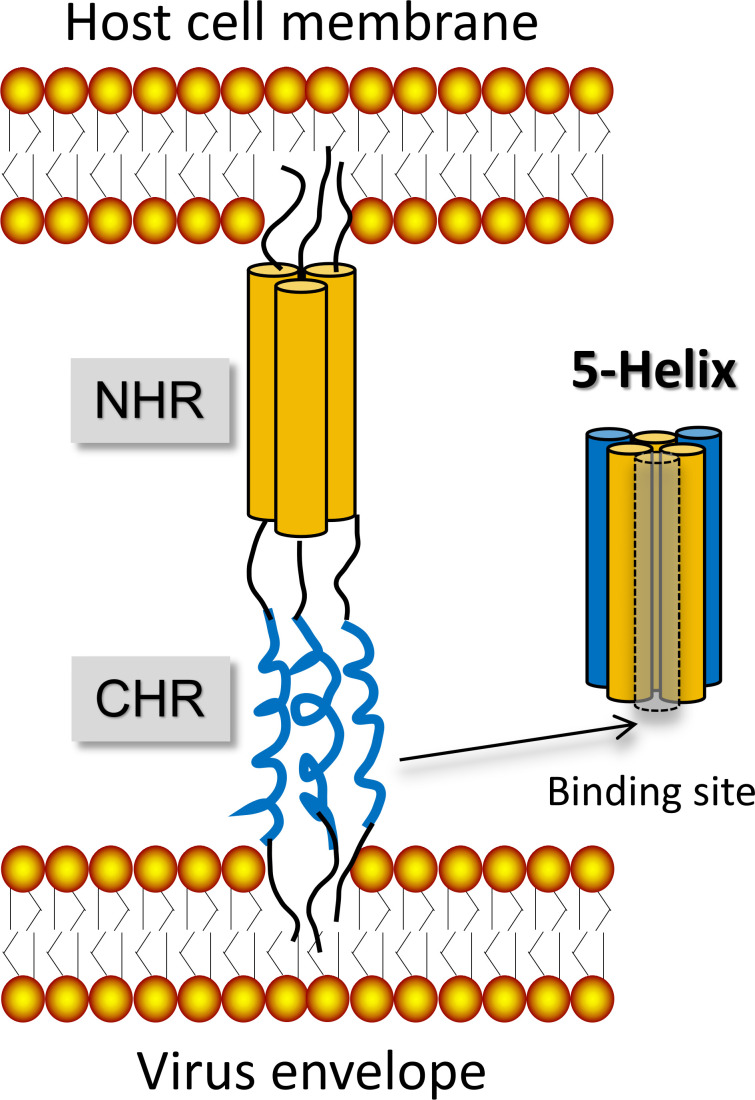
Schematic diagram of 5-Helix binding to the target.

Root et al. (55) designed a distinctive supercoiled protein, named 5-Helix, which is composed of three native N40 and two native C38. Arguably, 5-Helix is composed of three NHRs and two CHRs, which precisely provide a high-affinity binding site for the gp41 CHR region (Fig. 10). Under physiological conditions, 5-Helix is able to spontaneously fold into a pentamer that specifically binds to the CHR region to form the 6-HB mimics, which exhibit nanomolar levels of antiviral activity for inhibition of HIV-1 envelope-mediated cell-cell fusion. Through biotechnological recombinant techniques, peptides can be upgraded into macromolecular proteins that contain N-peptide sequences, thereby effectively exerting antiviral activity. The ability to artificially create proteins containing the N-trimer structure by biological recombination is an emerging strategy in recent years, which has been applied in the design of peptide-based fusion inhibitors for EBOV, MERS-CoV, and SARS-CoV-2, etc. (56–58).

## SUMMARY AND PROSPECT

Over the years, the key peptides to block the early membrane fusion process of HIV-1 invasion into host cells have made significant progress, thanks to the efforts of researchers who have gone before and after (59, 60). At present, peptide-based fusion inhibitors have also been intensively studied in other viruses such as SARS-CoV-2, MERS-CoV, and IAVs, etc., and even the peptide EK1 has been entered into the clinic against SARS-CoV-2, thereby demonstrating broad development prospects and significant application significance. We need to discover more and more peptide-based new chemical entities and establish entirely novel R&D strategies to lay the foundation for the future of peptide-based fusion inhibitors. Given the difficulties in the clinical application of current fusion inhibitors (such as T20), N-peptides with a unique active structure act on the target to exhibit excellent antiviral activity, serving as the main focus for future development with high expectations. This article summarizes the research strategies for novel N-peptides to date, offering valuable insights to researchers in this field. We also call for more researchers to join the peptide-based fusion inhibitors R&D in the future to support the treatment of viral infectious diseases.

## References

[1] Ashkenazi A, Shai Y. 2011. Insights into the mechanism of HIV-1 envelope induced membrane fusion as revealed by its inhibitory peptides. Eur Biophys J 40:349–357. doi:10.1007/s00249-010-0666-z 21258789

[2] Badani H, Garry RF, Wimley WC. 2014. Peptide entry inhibitors of enveloped viruses: the importance of interfacial hydrophobicity. Biochim Biophys Acta 1838:2180–2197. doi:10.1016/j.bbamem.2014.04.015 24780375 PMC7094693

[3] Swinkels HM, Justiz Vaillant AA, Nguyen AD, Gulick PG. 2024. HIV and AIDS. StatPearls, Treasure Island (FL), ineligible companies.Disclosure: Angel Justiz Vaillant declares no relevant financial relationships with ineligible companies. Disclosure: Andrew Nguyen declares no relevant financial relationships with ineligible companies. Disclosure: Peter Gulick declares no relevant financial relationships with ineligible companies.

[4] Hatfull GF, Dedrick RM, Schooley RT. 2022. Phage therapy for antibiotic-resistant bacterial infections. Annu Rev Med 73:197–211. doi:10.1146/annurev-med-080219-122208 34428079

[5] Hardy H, Skolnik PR. 2004. Enfuvirtide, a new fusion inhibitor for therapy of human immunodeficiency virus infection. Pharmacotherapy 24:198–211. doi:10.1592/phco.24.2.198.33141 14998221

[6] Luo H, Zhao Y, Ma Y, Liang G, Ga L, Meng Z. 2024. Design of artificial C-peptides as potential anti-HIV-1 inhibitors based on 6-HB formation mechanism. Protein Pept Lett 31:447–457. doi:10.2174/0109298665312274240530060233 38910421

[7] Wang H, Wang C. 2022. Peptide-based dual HIV and coronavirus entry inhibitors. Adv Exp Med Biol 1366:87–100. doi:10.1007/978-981-16-8702-0_6 35412136

[8] Xiao T, Cai Y, Chen B. 2021. HIV-1 entry and membrane fusion inhibitors. Viruses 13:735. doi:10.3390/v13050735 33922579 PMC8146413

[9] Champagne K, Shishido A, Root MJ. 2009. Interactions of HIV-1 inhibitory peptide T20 with the gp41 N-HR coiled coil. J Biol Chem 284:3619–3627. doi:10.1074/jbc.M809269200 19073602 PMC2635040

[10] Pan C, Liu S, Jiang S. 2010. HIV-1 gp41 fusion intermediate: a target for HIV therapeutics. J Formos Med Assoc 109:94–105. doi:10.1016/S0929-6646(10)60029-0 20206833

[11] He Y, Xiao Y, Song H, Liang Q, Ju D, Chen X, Lu H, Jing W, Jiang S, Zhang L. 2008. Design and evaluation of sifuvirtide, a novel HIV-1 fusion inhibitor. J Biol Chem 283:11126–11134. doi:10.1074/jbc.M800200200 18303020

[12] Lu J, Deeks SG, Hoh R, Beatty G, Kuritzkes BA, Martin JN, Kuritzkes DR. 2006. Rapid emergence of enfuvirtide resistance in HIV-1-infected patients: results of a clonal analysis. J Acquir Immune Defic Syndr 43:60–64. doi:10.1097/01.qai.0000234083.34161.55 16885776

[13] Mink M, Mosier SM, Janumpalli S, Davison D, Jin L, Melby T, Sista P, Erickson J, Lambert D, Stanfield-Oakley SA, Salgo M, Cammack N, Matthews T, Greenberg ML. 2005. Impact of human immunodeficiency virus type 1 gp41 amino acid substitutions selected during enfuvirtide treatment on gp41 binding and antiviral potency of enfuvirtide in vitro. J Virol 79:12447–12454. doi:10.1128/JVI.79.19.12447-12454.2005 16160172 PMC1211558

[14] Chan DC, Fass D, Berger JM, Kim PS. 1997. Core structure of gp41 from the HIV envelope glycoprotein. Cell 89:263–273. doi:10.1016/s0092-8674(00)80205-6 9108481

[15] Chong H, Wu X, Su Y, He Y. 2016. Development of potent and long-acting HIV-1 fusion inhibitors. AIDS 30:1187–1196. doi:10.1097/QAD.0000000000001073 26919736

[16] Chong H, Yao X, Zhang C, Cai L, Cui S, Wang Y, He Y. 2012. Biophysical property and broad anti-HIV activity of albuvirtide, a 3-maleimimidopropionic acid-modified peptide fusion inhibitor. PLoS One 7:e32599. doi:10.1371/journal.pone.0032599 22403678 PMC3293837

[17] Martin-Carbonero L. 2004. Discontinuation of the clinical development of fusion inhibitor T-1249. AIDS Rev 6:61. 15168742

[18] Suzuki K, Hiroaki H, Kohda D, Tanaka T. 1998. An isoleucine zipper peptide forms a native-like triple stranded coiled coil in solution. Protein Eng 11:1051–1055. doi:10.1093/protein/11.11.1051 9876926

[19] Eckert DM, Kim PS. 2001. Design of potent inhibitors of HIV-1 entry from the gp41 N-peptide region. Proc Natl Acad Sci U S A 98:11187–11192. doi:10.1073/pnas.201392898 11572974 PMC58705

[20] Chen X, Lu L, Qi Z, Lu H, Wang J, Yu X, Chen Y, Jiang S. 2010. Novel recombinant engineered gp41 N-terminal heptad repeat trimers and their potential as anti-HIV-1 therapeutics or microbicides. J Biol Chem 285:25506–25515. doi:10.1074/jbc.M110.101170 20538590 PMC2919114

[21] Na H, Liang G, Lai W. 2023. Isopeptide bond bundling superhelix for designing antivirals against enveloped viruses with class I fusion proteins: a review. CPB 24:1774–1783. doi:10.2174/1389201024666230330083640 37005549

[22] Wang C, Lai W, Yu F, Zhang T, Lu L, Jiang X, Zhang Z, Xu X, Bai Y, Jiang S, Liu K. 2015. De novo design of isopeptide bond-tethered triple-stranded coiled coils with exceptional resistance to unfolding and proteolysis: implication for developing antiviral therapeutics. Chem Sci 6:6505–6509. doi:10.1039/c5sc02220g 30090269 PMC6054081

[23] Wang B, Gallolu Kankanamalage S, Dong J, Liu Y. 2021. Optimization of therapeutic antibodies. Antib Ther 4:45–54. doi:10.1093/abt/tbab003 33928235 PMC7944496

[24] Bewley CA, Louis JM, Ghirlando R, Clore GM. 2002. Design of a novel peptide inhibitor of HIV fusion that disrupts the internal trimeric coiled-coil of gp41. J Biol Chem 277:14238–14245. doi:10.1074/jbc.M201453200 11859089

[25] Dwyer JJ, Wilson KL, Martin K, Seedorff JE, Hasan A, Medinas RJ, Davison DK, Feese MD, Richter HT, Kim H, Matthews TJ, Delmedico MK. 2008. Design of an engineered N-terminal HIV-1 gp41 trimer with enhanced stability and potency. Protein Sci 17:633–643. doi:10.1110/ps.073307608 18359857 PMC2271165

[26] Banach BB, Tripathi P, Da Silva Pereira L, Gorman J, Nguyen TD, Dillon M, Fahad AS, Kiyuka PK, Madan B, Wolfe JR, et al.. 2022. Highly protective antimalarial antibodies via precision library generation and yeast display screening. J Exp Med 219:e20220323. doi:10.1084/jem.20220323 35736810 PMC9242090

[27] Wu W, Lin D, Shen X, Li F, Fang Y, Li K, Xun T, Yang G, Yang J, Liu S, He J. 2015. New influenza A virus entry inhibitors derived from the viral fusion peptides. PLoS ONE 10:e0138426. doi:10.1371/journal.pone.0138426 26382764 PMC4575187

[28] Xia S, Yan L, Xu W, Agrawal AS, Algaissi A, Tseng C-TK, Wang Q, Du L, Tan W, Wilson IA, Jiang S, Yang B, Lu L. 2019. A pan-coronavirus fusion inhibitor targeting the HR1 domain of human coronavirus spike. Sci Adv 5:eaav4580. doi:10.1126/sciadv.aav4580 30989115 PMC6457931

[29] Simm D, Hatje K, Waack S, Kollmar M. 2021. Critical assessment of coiled-coil predictions based on protein structure data. Sci Rep 11:12439. doi:10.1038/s41598-021-91886-w 34127723 PMC8203680

[30] Apostolovic B, Danial M, Klok HA. 2010. Coiled coils: attractive protein folding motifs for the fabrication of self-assembled, responsive and bioactive materials. Chem Soc Rev 39:3541–3575. doi:10.1039/b914339b 20676430

[31] Fletcher JM, Boyle AL, Bruning M, Bartlett GJ, Vincent TL, Zaccai NR, Armstrong CT, Bromley EHC, Booth PJ, Brady RL, Thomson AR, Woolfson DN. 2012. A basis set of de novo coiled-coil peptide oligomers for rational protein design and synthetic biology. ACS Synth Biol 1:240–250. doi:10.1021/sb300028q 23651206

[32] Eckert DM, Malashkevich VN, Kim PS. 1998. Crystal structure of GCN4-pIQI, a trimeric coiled coil with buried polar residues. J Mol Biol 284:859–865. doi:10.1006/jmbi.1998.2214 9837709

[33] Papanikolopoulou K, Forge V, Goeltz P, Mitraki A. 2004. Formation of highly stable chimeric trimers by fusion of an adenovirus fiber shaft fragment with the foldon domain of bacteriophage t4 fibritin. J Biol Chem 279:8991–8998. doi:10.1074/jbc.M311791200 14699113

[34] Yang X, Lee J, Mahony EM, Kwong PD, Wyatt R, Sodroski J. 2002. Highly stable trimers formed by human immunodeficiency virus type 1 envelope glycoproteins fused with the trimeric motif of T4 bacteriophage fibritin. J Virol 76:4634–4642. doi:10.1128/jvi.76.9.4634-4642.2002 11932429 PMC155086

[35] Clinton TR, Weinstock MT, Jacobsen MT, Szabo-Fresnais N, Pandya MJ, Whitby FG, Herbert AS, Prugar LI, McKinnon R, Hill CP, Welch BD, Dye JM, Eckert DM, Kay MS. 2015. Design and characterization of ebolavirus GP prehairpin intermediate mimics as drug targets. Protein Sci 24:446–463. doi:10.1002/pro.2578 25287718 PMC4380977

[36] Bi W, Chen G, Dang B. 2022. Novel engineered SARS-CoV-2 HR1 trimer exhibits improved potency and broad-spectrum activity against SARS-CoV-2 and Its Variants. J Virol 96:e0068122. doi:10.1128/jvi.00681-22 35735997 PMC9278106

[37] Song A, Yu H, Wang C, Zhu X, Liu K, Ma X. 2015. Novel dual small-molecule HIV inhibitors: scaffolds and discovery strategies. Curr Pharm Des 21:950–962. doi:10.2174/1381612820666140929095102 25269561

[38] Nakahara T, Nomura W, Ohba K, Ohya A, Tanaka T, Hashimoto C, Narumi T, Murakami T, Yamamoto N, Tamamura H. 2010. Remodeling of dynamic structures of HIV-1 envelope proteins leads to synthetic antigen molecules inducing neutralizing antibodies. Bioconjug Chem 21:709–714. doi:10.1021/bc900502z 20359196

[39] Wu C, Raheem IT, Nahas DD, Citron M, Kim PS, Montefiori DC, Ottinger EA, Hepler RW, Hrin R, Patel SB, Soisson SM, Joyce JG. 2024. Stabilized trimeric peptide immunogens of the complete HIV-1 gp41 N-heptad repeat and their use as HIV-1 vaccine candidates. Proc Natl Acad Sci U S A 121:e2317230121. doi:10.1073/pnas.2317230121 38768344 PMC11145295

[40] Nomura W, Mizuguchi T, Tamamura H. 2016. Multimerized HIV-gp41-derived peptides as fusion inhibitors and vaccines. Biopolymers 106:622–628. doi:10.1002/bip.22782 26583370

[41] Bai Y, Xue H, Wang K, Cai L, Qiu J, Bi S, Lai L, Cheng M, Liu S, Liu K. 2013. Covalent fusion inhibitors targeting HIV-1 gp41 deep pocket. Amino Acids 44:701–713. doi:10.1007/s00726-012-1394-8 22961335

[42] Lai W, Wang C, Yan J, Liu H, Zhang W, Lin B, Xi Z. 2020. Suitable fusion of N-terminal heptad repeats to achieve covalently stabilized potent N-peptide inhibitors of HIV-1 infection. Bioorg Med Chem 28:115214. doi:10.1016/j.bmc.2019.115214 31932193

[43] Louis JM, Nesheiwat I, Chang L, Clore GM, Bewley CA. 2003. Covalent trimers of the internal N-terminal trimeric coiled-coil of gp41 and antibodies directed against them are potent inhibitors of HIV envelope-mediated cell fusion. J Biol Chem 278:20278–20285. doi:10.1074/jbc.M301627200 12654905

[44] Bianchi E, Finotto M, Ingallinella P, Hrin R, Carella AV, Hou XS, Schleif WA, Miller MD, Geleziunas R, Pessi A. 2005. Covalent stabilization of coiled coils of the HIV gp41 N region yields extremely potent and broad inhibitors of viral infection. Proc Natl Acad Sci U S A 102:12903–12908. doi:10.1073/pnas.0502449102 16129831 PMC1200264

[45] Kobayakawa T, Ebihara K, Honda Y, Fujino M, Nomura W, Yamamoto N, Murakami T, Tamamura H. 2019. Dimeric C34 derivatives linked through disulfide bridges as new HIV-1 fusion inhibitors. Chembiochem 20:2101–2108. doi:10.1002/cbic.201900187 31012222

[46] Kang HJ, Baker EN. 2011. Intramolecular isopeptide bonds: protein crosslinks built for stress? Trends Biochem Sci 36:229–237. doi:10.1016/j.tibs.2010.09.007 21055949

[47] Pickart CM. 2001. Mechanisms underlying ubiquitination. Annu Rev Biochem 70:503–533. doi:10.1146/annurev.biochem.70.1.503 11395416

[48] Schoene C, Fierer JO, Bennett SP, Howarth M. 2014. SpyTag/SpyCatcher cyclization confers resilience to boiling on a mesophilic enzyme. Angew Chem Int Ed Engl 53:6101–6104. doi:10.1002/anie.201402519 24817566 PMC4286826

[49] Veggiani G, Zakeri B, Howarth M. 2014. Superglue from bacteria: unbreakable bridges for protein nanotechnology. Trends Biotechnol 32:506–512. doi:10.1016/j.tibtech.2014.08.001 25168413 PMC4281928

[50] Lai W, Wang C, Yu F, Lu L, Wang Q, Jiang X, Xu X, Zhang T, Wu S, Zheng X, Zhang Z, Dong F, Jiang S, Liu K. 2016. An effective strategy for recapitulating N-terminal heptad repeat trimers in enveloped virus surface glycoproteins for therapeutic applications. Chem Sci 7:2145–2150. doi:10.1039/c5sc04046a 29899942 PMC5968561

[51] Wang C, Li X, Yu F, Lu L, Jiang X, Xu X, Wang H, Lai W, Zhang T, Zhang Z, Ye L, Jiang S, Liu K. 2016. Site-specific isopeptide bridge tethering of chimeric gp41 N-terminal heptad repeat helical trimers for the treatment of HIV-1 infection. Sci Rep 6:32161. doi:10.1038/srep32161 27562370 PMC4999862

[52] Huang Y, Luo H, Jin Y, Ma Y, Zhao Y, Gao X, Zhao Y, Qi X, Liang G, Ga L, Li G, Yang J. 2024. Design of coiled-coil N-peptides against HIV-1 based on a CADD strategy. Org Biomol Chem 23:157–166. doi:10.1039/d4ob01620c 39523986

[53] Krainer FW, Glieder A. 2015. An updated view on horseradish peroxidases: recombinant production and biotechnological applications. Appl Microbiol Biotechnol 99:1611–1625. doi:10.1007/s00253-014-6346-7 25575885 PMC4322221

[54] Louis JM, Bewley CA, Gustchina E, Aniana A, Clore GM. 2005. Characterization and HIV-1 fusion inhibitory properties of monoclonal Fabs obtained from a human non-immune phage library selected against diverse epitopes of the ectodomain of HIV-1 gp41. J Mol Biol 353:945–951. doi:10.1016/j.jmb.2005.09.044 16216270

[55] Root MJ, Kay MS, Kim PS. 2001. Protein design of an HIV-1 entry inhibitor. Science 291:884–888. doi:10.1126/science.1057453 11229405

[56] Harrison JS, Higgins CD, Chandran K, Lai JR. 2011. Designed protein mimics of the Ebola virus glycoprotein GP2 α-helical bundle: stability and pH effects. Protein Sci 20:1587–1596. doi:10.1002/pro.688 21739501 PMC3190153

[57] Xing L, Xu X, Xu W, Liu Z, Shen X, Zhou J, Xu L, Pu J, Yang C, Huang Y, Lu L, Jiang S, Liu S. 2022. A five-helix-based SARS-CoV-2 fusion inhibitor targeting heptad repeat 2 domain against SARS-CoV-2 and its variants of concern. Viruses 14:597. doi:10.3390/v14030597 35337003 PMC8955665

[58] Sun Y, Zhang H, Shi J, Zhang Z, Gong R. 2017. Identification of a novel inhibitor against Middle East respiratory syndrome coronavirus. Viruses 9:255. doi:10.3390/v9090255 28906430 PMC5618021

[59] Malik T, Chauhan G, Rath G, Murthy RSR, Goyal AK. 2017. “Fusion and binding inhibition” key target for HIV-1 treatment and pre-exposure prophylaxis: targets, drug delivery and nanotechnology approaches. Drug Deliv 24:608–621. doi:10.1080/10717544.2016.1228717 28240046 PMC8241151

[60] Pu J, Zhou JT, Liu P, Yu F, He X, Lu L, Jiang S. 2022. Viral entry inhibitors targeting six-helical bundle core against highly pathogenic enveloped viruses with class I fusion proteins. CMC 29:700–718. doi:10.2174/0929867328666210511015808 33992055

